# Ether and Chloroform

**Published:** 1848-04

**Authors:** 


### ETHER AND CHLOROFORM.

These anæsthetic agents, both of which in the last few months have been extensively used in surgical operations, are likely to be used to considerable extent as remedial agents for various diseases.

We see reported in the Missouri Medical and Surgical Journal a case of delirium tremens, treated by the inhalation of Ether, with apparent good success, by W. C. Anderson, M. D., *Seamen's Retreat,* Staten Island; and we find that some of our first physicians of this city are administering Chloroform internally in nervous and spasmodic affections, and also externally in local affections of the skin, &c.

The case of death reported in this number of the Register, as the result of Chloroform inhalation, has given quite a check to its use in dental operations. But to throw aside every remedial agent which may or has produced death, would be to deprive us of some of our most valuable remedies for the cure of disease. It should, however, make us careful in the use of such agents, and if properly improved, such lessons may direct us so to use them, as that they shall prove of incalculable benefit to our race.

We have seen marked benefit from the use of both these articles, and we have seen them used when it would have been better to have operated without them. Persons who want but one or two teeth removed can usually have it done without the aid of any such remedies. The operation would produce less prostration of the system than the free use of either of these agents.

We think that any agent possessing sufficient power to, in so short a time, deprive us of consciousness and sensibility, must be capable, if carried beyond a certain point, to produce fatal results.

It becomes us, then, to know when and to what extent these agents may be made useful—time and close observation will determine this.—CIN. ED.

